# Effect of context on respiratory rate measurement in identifying non‐severe pneumonia in African children

**DOI:** 10.1111/tmi.12492

**Published:** 2015-03-27

**Authors:** Florida Muro, George Mtove, Neema Mosha, Hannah Wangai, Nicole Harrison, Helena Hildenwall, David Schellenberg, Jim Todd, Raimos Olomi, Hugh Reyburn

**Affiliations:** ^1^ Kilimanjaro Christian Medical University CollegeMoshiTanzania; ^2^ Teule HospitalMuhezaTanzania; ^3^ Joint Malaria ProgrammeKilimanjaro Christian Medical CentreMoshiTanzania; ^4^ Division of Infectious Diseases and Tropical MedicineMedical University of ViennaViennaAustria; ^5^ London School of Hygiene & Tropical MedicineLondonUK; ^6^ Department of Public Health SciencesKarolinska InstitutetStockholmSweden

**Keywords:** pneumonia, respiratory rate, children, Integrated Management of Childhood Illness, Africa, pneumonie, fréquence respiratoire, enfants, PCIME, Afrique

## Abstract

**Objective:**

Cough or difficult breathing and an increased respiratory rate for their age are the commonest indications for outpatient antibiotic treatment in African children. We aimed to determine whether respiratory rate was likely to be transiently raised by a number of contextual factors in a busy clinic leading to inaccurate diagnosis.

**Methods:**

Respiratory rates were recorded in children aged 2–59 months presenting with cough or difficulty breathing to one of the two busy outpatient clinics and then repeated at 10‐min intervals over 1 h in a quiet setting.

**Results:**

One hundred and sixty‐seven children were enrolled with a mean age of 7.1 (SD ± 2.9) months in infants and 27.6 (SD ± 12.8) months in children aged 12–59 months. The mean respiratory rate declined from 42.3 and 33.6 breaths per minute (bpm) in the clinic to 39.1 and 32.6 bpm after 10 min in a quiet room and to 39.2 and 30.7 bpm (*P* < 0.001) after 60 min in younger and older children, respectively. This resulted in 11/13 (85%) infants and 2/15 (13%) older children being misclassified with non‐severe pneumonia. In a random effects linear regression model, the variability in respiratory rate within children (42%) was almost as much as the variability between children (58%). Changing the respiratory rates cut‐offs to higher thresholds resulted in a small reduction in the proportion of non‐severe pneumonia mis‐classifications in infants.

**Conclusion:**

Noise and other contextual factors may cause a transient increase in respiratory rate and consequently misclassification of non‐severe pneumonia. However, this effect is less pronounced in older children than infants. Respiratory rate is a difficult sign to measure as the variation is large between and within children. More studies of the accuracy and utility of respiratory rate as a proxy for non‐severe pneumonia diagnosis in a busy clinic are needed.

## Abstract (fr)

**Objectif:**

La toux ou la respiration difficile et une augmentation de la fréquence respiratoire pour leur âge sont les plus fréquentes indications pour le traitement ambulatoire aux antibiotiques chez les enfants africains. Nous avons cherché à déterminer s'il était possible que la fréquence respiratoire soit transitoirement augmentée par un certain nombre de facteurs contextuels dans une clinique surchargée, menant à un diagnostic inexact.

**Méthodes:**

Les fréquences respiratoires ont été enregistrées chez des enfants âgés de 2 à 59 mois présentant une toux ou des difficultés respiratoires dans l'une des deux cliniques ambulatoires surchargées et ensuite répétées à intervalles de 10 minutes pendant 1 heure dans un endroit calme.

**Résultats:**

167 enfants ont été investigués avec un âge moyen de 7,1 (SD ± 2,9) mois pour les nourrissons et 27,6 (SD ± 12,8) mois pour ceux âgés de 12 à 59 mois. La fréquence respiratoire moyenne a baissée de 42,3 et 33,6 respirations par minute (bpm) dans la clinique à 39,1 et 32,6 bpm après 10 minutes dans une chambre calme et à 39,2 et 30,7 bpm (p < 0,001) après 60 minutes chez les plus jeunes enfants et les plus âgés respectivement. Cela a résulté en 11/13 (85%) nourrissons et 2/15 (13%) enfants plus âgés classés erronément comme ayant une pneumonie non sévère. Dans un modèle de régression linéaire à effets aléatoires, la variabilité de la fréquence respiratoire chez un enfant (42%) était presque autant que la variabilité entre enfants (58%). La modification des seuils de la fréquence respiratoire à des seuils plus élevés a entraîné une légère réduction de la proportion de cas de pneumonie non sévère erronément classifiés chez les nourrissons.

**Conclusion:**

Le bruit et d'autres facteurs contextuels peuvent provoquer une augmentation transitoire de la fréquence respiratoire et par conséquent une mauvaise classification de la pneumonie non sévère. Cependant, cet effet est moins prononcé chez les enfants plus âgés que chez les nourrissons. La fréquence respiratoire est un signe difficile à mesurer car la variabilité est grande chez un enfant et entre enfants. Des études supplémentaires sur l'exactitude et l'utilité de la fréquence respiratoire comme proxy pour le diagnostic de la pneumonie non sévère dans une clinique surchargée sont nécessaires.

## Abstract (es)

**Objetivo:**

La tos o la dificultad respiratoria y una frecuencia respiratoria aumentada para la edad son la indicación más común para recetar tratamiento antibiótico en consultas externas a niños Africanos. Buscábamos determinar si la frecuencia respiratoria podría estar transitoriamente aumentada por un número de factores contextuales tales como una clínica con mucho movimiento, con el resultado de un diagnóstico inexacto.

**Métodos:**

Se anotaron las frecuencias respiratorias de niños con edades entre 2–59 meses que se presentaron con tos o dificultad respiratoria en uno o dos de los centros de consultas externas con mucho movimiento y después se repitieron en intervalos de 10 minutos, durante 1 hora y en un lugar tranquilo.

**Resultados:**

Se incluyeron 167 niños con una edad media de 7.1 (SD ± 2.9) meses para los lactantes y de 27.6 (SD ± 12.8) meses para niños con edades entre 12–59 meses. La frecuencia respiratoria media disminuyó de 42.3 y 33.6 respiraciones por minuto (rpm) en el centro sanitario a 39.1 y 32.6 rpm después de 10 minutos en una habitación tranquila y a 39.2 y 30.7 rpm (p < 0.001) después de 60 minutos en lactantes y niños respectivamente. Esto resultó en que 11/13 (85%) lactantes y 2/15 (13%) niños fuesen clasificados erróneamente con neumonía no severa. En un modelo de regresión lineal de efectos aleatorios la variabilidad en la frecuencia respiratoria de un mismo niño (42%) era casi tanta como la variabilidad entre los niños (58%). Los cambios en los puntos de corte de las frecuencias respiratorias a límites más altos resultaron en una reducción pequeña en la proporción de neumonía no severa mal clasificada entre niños.

**Conclusión:**

El ruido y otros factores contextuales pueden causar un aumento transitorio en la frecuencia respiratoria y como consecuencia en una clasificación errónea de la neumonía no severa. Sin embargo, este efecto es menos pronunciado en niños mayores que entre lactantes. La tasa respiratoria es un signo difícil de medir puesto que la variación es grande en un mismo niño y entre niños. Se requieren más estudios sobre la precisión y utilidad de la frecuencia respiratoria como un signo para el diagnóstico de la neumonía no severa en un centro sanitario con mucho movimiento.

## Introduction

Pneumonia is the leading cause of death in young children causing an estimated 1.2 million deaths in children aged under 5 years worldwide, over 90% of which occur in resource‐poor countries 1, 2, 3. In Africa, the incidence of pneumonia in young children has been estimated at 0.28 episodes/child‐year and an estimated 32% of these are due to bacterial infections where progression from early stages of pneumonia to severe and potentially fatal pneumonia might be prevented by antibiotic treatment taken early in the illness 4, 5. A number of studies have demonstrated the association between raised respiratory rate (RR) and severe pneumonia, a finding that underpins the WHO‐Integrated Management of Childhood Illness (IMCI) recommendation that children under the age of 5 years presenting with cough or difficult breathing and an increased RR for their age meet the IMCI criteria for ‘non‐severe pneumonia’ and should receive antibiotic treatment 6. This guideline recommends that RR be assessed in a calm child and for a whole minute (60 s). It is also possible to count the respiratory rate during sleeping or feeding as the high respiratory rates tend to persist in a child with pneumonia.

These IMCI strategies are appropriate to a scenario of high incidence of bacterial pneumonia, but a number of recent developments raise the need for a review. Firstly, the introduction of vaccines against the most common bacterial causes of pneumonia into resource‐poor countries (*Haemophilus influenza type b* (*Hib*) vaccine and *Streptococcus pneumoniae* conjugate vaccine) continues to result in large reductions in the incidence of bacterial pneumonia 7. Currently, 36 African countries have introduced *Hib* vaccine into the routine vaccination schedule, and in these countries, invasive disease due to this organism has almost disappeared 8, 9. The use of pneumococcal conjugate vaccine is more recent, but all but two African countries are committed to its introduction and early evidence suggests that it may contribute to a reduction in invasive disease 7, 10. Alongside these developments, the WHO recommendation for malaria diagnosis changed in 2010 from ‘presumptive treatment’ to the recommendation that wherever possible suspected malaria should be confirmed by a parasitological test before initiating treatment 11. This policy is being implemented through a large scale‐up in the use of rapid immunochromatographic tests and this is generating large numbers of children previously treated for malaria but now considered for a diagnosis of non‐severe pneumonia 12, 13. The consequent increase in the use of antibiotics that has been consistently observed following the introduction of RDTs raises concerns regarding selection pressure for resistance to affordable antimicrobial agents in Africa to which there is already evidence of reduced susceptibility 14, 15, 16.

Compounding the above considerations, measurement of RR in young children often lacks precision. Interobserver variation on RR reaches only moderate levels of agreement, and RR may be influenced by the presence of fever 17, 18, 19. It seems likely that RR in children is influenced by noise and anxiety often present in routine clinic settings. We thus conducted this study to investigate the effect of context in a busy clinic on the classification of non‐severe pneumonia in young children in Tanzania. We anticipate that such evidence will represent an important addition to the knowledge of the accuracy and limitations of respiratory rate measurements in guiding the use of antimicrobial agents in children.

## Methods

### Study area

The study was conducted in each of two outpatient departments of Teule district designated hospital, Muheza and St. Joseph Municipal Council designated hospital, Moshi North East Tanzania, the first serving a predominantly rural population exposed to moderate degrees of malaria transmission (parasite prevalence in children 14.5%) and the second an area of low malaria endemicity serving a predominantly urban population 20.

### Enrolment and clinical measurement

Children aged 2–59 months presenting with cough or difficulty in breathing were screened for the following inclusion criteria: negative to testing with Paracheck^™^ rapid test for malaria, Hb > 8 g/dl (Hemocue^™^), no use of anticonvulsant or sedative medication in the previous 24 h, no signs of severe illness including lower chest wall in‐drawing, no evidence of chronic illness including asthma and malnutrition. To maximise the accuracy of the medical history, children presented by a caretaker other than their biological mother were excluded.

After enrolment, a medical history and basic socio‐demographic details were collected, followed by a careful physical examination including measurement of respiratory rate over 1 min done by a well‐trained research nurse under the supervision of the clinician.

After initial assessment, children were transferred to a quiet room where they were observed by a research nurse for 1 h and RR repeated every 10 min using respiratory rate timer while also video recording the child's chest movement. During observation, the child's state was documented (awake/calm, sleeping, feeding and agitated) on the case report form. Agitation was defined as any movement and, or, crying that interrupted counting of the RR. In circumstances where children could not settle, counting of RR was stopped and repeated after another 10 min of rest.

### Data management and analysis

Data were double‐entered into Microsoft Access and statistical analyses performed using STATA version 11. The study participants were analysed in two age groups based on the WHO's IMCI cut‐offs for age, that is ≥50 breaths per minute (bpm) for those aged 2–12 months and ≥40 bpm for 12–59 months. Non‐severe pneumonia was defined according to WHO/IMCI as cough or difficulty breathing with a raised RR for age. Any other sign such as chest in‐drawing or general danger signs were considered severe and therefore not part of our inclusion criteria.

The respiratory rate was analysed for each time point in two ways: by considering each observation as independent, the mean respiratory rate at each time point was compared against the baseline measurement using Student's *t*‐test and by considering the observed respiratory rates were normally distributed around the mean for each child, we could develop a random effects linear regression model to explain differences in respiratory rates. In this model, we used fixed effects for child characteristics and the time characteristics and assessed the within‐child variability in respiratory rates after allowing for these effects. For all analyses, infants (<12 months) and children (aged 12–59 months) were considered separately.

Sensitivity, specificity and 95% confidence intervals were calculated for repeat respiratory rate (RR) measurements against the 10‐min RR as a reference measure.

### Ethics

This study was approved by the Kilimanjaro Christian Medical Centre (KCMC) Research Ethics Committee (Certificate No.481), and mothers of all children enrolled provided a written informed consent for participation.

## Results

### Screening of children in the study

**Figure 1:**
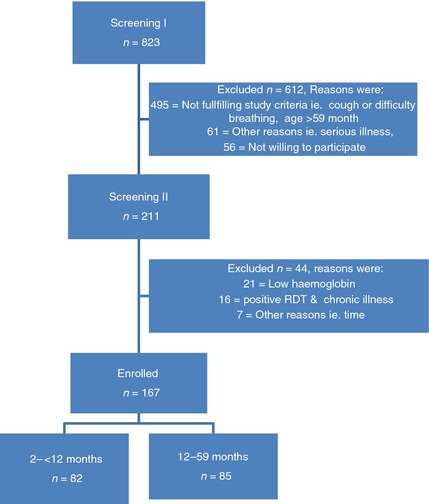
Flow chart representing the number of children screened and enrolled.

The numbers of children screened and enrolled are shown in Figure 1; of 823 children who were screened, 167 (20.3%) met study inclusion criteria and were enrolled. The commonest reasons for exclusion were being out of age range, no history of cough or difficulty in breathing or signs of severe illness. During the second stage of screening, 59 children were excluded due to chronic illness, refusal to participate or anaemia (Hb < 8 g/dl).

Enrolled children were approximately equally distributed between infants (2–11 months, *n* = 82) and older children (12–59 months, *n* = 85). Among infants 54 (65.9%) were male, while among older children 44 (50.6%) were male. The mean age of infants and older children was 7.1 months (SD ± 2.9) and 27.6 months (SD ± 12.8), respectively. Further details of participant characteristics are presented in Table 1.

**Table 1:** Baseline characteristics of the study children according to age group, *N* = 167

Characteristics	Infants (<12 month) (*n* = 82)	Older (≥12 month) (*n* = 85)
Sex		
Male *n* (%)	54 (65.85)	44 (50.59)
Age		
Mean age (SD) in months	7.1 (2.9)	27.6 (12.8)
Initial respiratory rate (RR) in bpm		
Mean (SD) RR	42.3 (0.88)	33.6 (1.03)
Median (IQR) RR	43 (36–48)	32 (27–37)
Normal (SD) RR	66 (80.49)	68 (80.00)
High (SD) RR	16 (19.51)	17 (20.00)
Mean (SD) temperature in °C	36.9 (0.71)	36.9 (0.84)
Mean (SD) haemoglobin in g/dL	10.2 (0.12)	11.0 (0.13)
Mean (SD) days ill	5.2 (10.58)	4.76 (4.86)
History of rapid breathing		
No	43 (52.44)	58 (68.24)
Yes	39 (47.56)	27 (31.76)
Pre‐consultation medication last 7 days		
No	31 (37.80)	31 (36.47)
Yes	51 (62.20)	54 (63.53)

### Respiratory rate measurements over time

**Figure 2:**
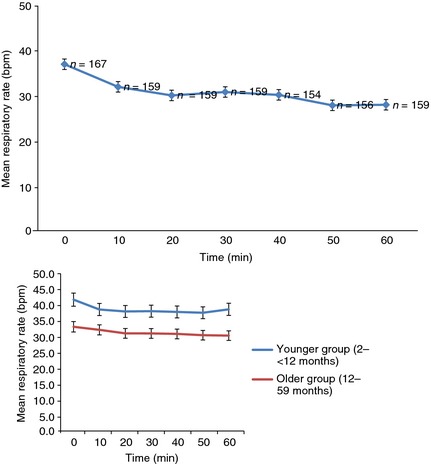
Mean respiratory rates measured by research nurse over 1 h according to age groups. Upper and lower limits denote 95% confidence intervals.

Figure 2 shows the mean respiratory rates observed by the clinician compared with those observed by the research nurse in the quiet room over the following 1 h.

For 144 children, comparison of the respiratory rates at initial (*t* = 0) and at 10 min (*t* = 10) showed that the respiratory rate was unchanged in 11 (7.6%) children and increased in 53(36.8%) children, of whom 11 (7.6%) were increased by more than 10 breaths per minute. There were 80 (55.6%) children with lower RR, of whom 23 (16.0%) were lower by more than 10 breaths per minute. Overall, the mean difference in respiratory rates at *t* = 10 compared to *t* = 0 was 1.8 bpm lower (95% CI, 0.32–3.20, paired *t*‐test = 2.41, *P* = 0.017). Similar decreases were shown in subsequent respiratory rate measures.

### Changes in proportion of children meeting criteria for non‐severe pneumonia (NSP) over time

There was a gradual decline in the proportion of children who met the criteria for non‐severe pneumonia over time, and this was more pronounced in the older age group compared to younger group. However, in a small proportion of children, respiratory rate (RR) increased in a quiet room compared with the rate observed in the clinic and this was more marked in infants (Table 2).

**Table 2:** The number of children satisfying the criteria for non‐severe pneumonia at enrolment and over the subsequent 60 min

Time	Younger children (RR ≥ 50 bpm) *N* = 16			Younger children (RR <50 bpm) *N* = 66			Older children (RR ≥ 40 bpm) *N* = 17			Older children (RR < 40 bpm) *N* = 68		
	Raised	Normal	Missing	Normal	Raised	Missing	Raised	Normal	Missing	Normal	Raised	Missing
0	16	0	0	66	0	0	17	0	0	68	0	0
10	2	11	3	47	10	9	13	2	2	54	5	8
20	3	8	5	49	7	10	8	5	4	55	4	8
30	3	9	4	54	4	8	7	5	5	55	5	8
40	3	9	4	46	11	9	4	7	6	54	3	11
50	3	8	5	52	4	10	5	4	8	46	5	17
60	4	8	4	47	6	13	3	6	8	50	3	15

Proportion of children who never, once or always met criteria for non‐severe pneumonia in the hour following enrolment				
Never	10 (62.5%)	47 (71.2%)	4 (23.5%)	59 (86.8%)
Once	2 (12.5%)	9 (13.6%)	2 (11.8%)	2 (2.9%)
>Once	4 (25.0%)	10 (15.2%)	11 (64.7%)	7 (10.3%)

### Sensitivity and Specificity

Using the 10‐min respiratory rate in the quiet room as a reference, the sensitivity was generally lower in both age groups; 16.7% in infants and 72.2% in older children. However, specificity was good, 81% and 96.4%, respectively.

### Sensitivity analysis

**Figure 3:**
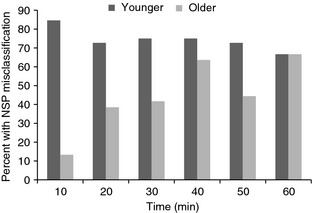
Proportion of children misclassified for non‐severe pneumonia (NSP) at different time intervals.

**Figure 4:**
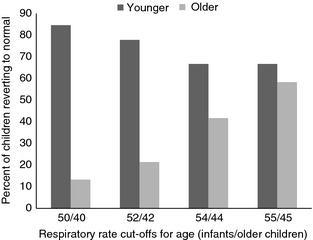
Proportion of children whose respiratory rate became normal at 10** **min of rest; now using different cut‐off levels of respiratory rate (RR).

We hypothesised that variation in the proportion of children meeting non‐severe pneumonia diagnostic criteria might decline over time if different cut‐offs of respiratory rates were applied. The proportion of children misclassified for non‐severe pneumonia (in subsequent time periods following the first classification) declined as the cut‐off was increased, while among older children, there was only slight increase (Figure 3 and 4).

### Regression model using baseline and subsequent RR measures

A random effects linear regression was used to characterise the association of the respiratory rate of the child and clinical characteristics of sex, age, temperature at baseline, receipt of medication within the previous 7 days and different child's state during RR measurement (i.e. awake/calm, sleeping, feeding and agitated). The variability within children was 58% and that between children 42% (Table 3).

**Table 3:** Multivariate random effects linear regressions taking all respiratory rate observations over the 60 min

Variable	Coefficient	Standard error (SE)	95% Confidence interval	*P*‐Value
Female	0.28	1.12	−1.91: 2.4	0.802
Age (as continuous)	−0.29	0.06	−0.41: −0.18	**<0.001**
Temperature (°C) at baseline	4.99	0.73	3.57: 6.43	**<0.001**
Days ill (Continuous)	−0.12	0.07	−0.25: −0.01	0.077
History of rapid breathing				
Yes	1.04	1.13	−0.18: 3.26	0.358
Received med past 7 days				
Yes	1.98	1.48	−0.93: 4.89	0.183
Child's state				
Awake/calm	−1.16	0.64	−2.42: −0.09	0.071
Sleeping	−6.18	0.84	−7.82: −4.53	**<0.001**
Agitated	−3.32	0.82	−4.94: −1.71	**<0.001**
Feeding	0.89	1.16	−1.38: 3.16	0.41
Baseline	Base			
Time	0.00	0.01	−0.02: 0.02	0.947

Final model included sex, age, temperature at baseline, number of days ill prior to presentation at the clinic, history of rapid breathing, whether the child received medication and the child's state at the time of measuring the respiratory rate.

Within cluster variation = 6.52; Between cluster variation = 5.56; Intraclass variation = 58%.

Bold p‐values indicates significant associations in multivariate analysis.

## Discussion

The study has confirmed that children's respiratory rate is sensitive to the conditions of noise and disturbance that are often present in busy clinics in Africa. The clustering of respiratory rate readings close to the age‐specific cut‐offs for identifying non‐severe pneumonia resulted in substantial misclassification of children. This effect varied by age whereby the proportion of children misclassified as having non‐severe pneumonia at the initial examination compared to subsequent examinations was more pronounced in infants than in older children. The reasons for this difference are not clear, but it seems possible that older children are more able to rationalise the disturbances around them, while younger children may react more instinctively.

Although there was a general tendency for respiratory rate to settle during the first 10 min in quiet surroundings, a minority of children's respiratory rate actually increased. This was found in both infants and older children and may indicate either a random variation in respiratory rates over relatively short periods of time or inaccuracies in measurement.

Although repeated measurements of respiratory rate can improve reliability of the sign as was shown in our study and others, it is, however, unrealistic in a busy clinic and there are a number of studies that have already documented that healthcare workers appear to be relatively reluctant to use of respiratory rate in their routine practice and that RR is rarely recorded 21, 22, 23, 24, 25.

We hypothesised that small increases in the diagnostic cut‐off that define ‘non‐severe pneumonia’ might reduce the level of misclassification, but this did not appear to make a substantial difference. Again, this may be due to a degree of random variation in respiratory rate in children over relatively short periods of time that counter‐balanced the systematic reduction in respiratory rate associated with settling a child in a quiet environment. Over 40% of the observed variability in respiratory rates was seen within children over a relatively short period of time (60 min). Almost a quarter of children had a change in respiratory rate of 10 breaths per minute within 10 min, making the use of any cut‐offs unreproducible and possibly unreliable.

### The association between respiratory rate and pneumonia

A number of studies have documented an association between respiratory rate and severe pneumonia 26, 27. A study of children attending the paediatric emergency department in Boston, USA, found that the presence of tachypnoea with no other abnormal respiratory signs did not predict the presence of radiological pneumonia 28. More importantly, a placebo‐controlled trial of amoxicillin conducted in Pakistan failed to demonstrate a benefit of amoxycillin over placebo in children with IMCI criteria of non‐severe pneumonia 29. However, other studies suggest that the aetiology and clinical course of pneumonia in children may vary considerably between Africa and Asia 30. Our study results indicate a decline in the number of children who will require antibiotics according to the IMCI guideline if they were kept in a calm environment for a longer period of time. In the age of vaccines against bacterial pneumonia and widespread use of parasitological testing for malaria, there is a need for a placebo‐controlled trial of amoxicillin in African children meeting criteria of non‐severe pneumonia who also undergo a radiological examination to assist diagnosis. The results of such a trial may provide an opportunity to reduce antibiotic use in Africa and could also question the utility of respiratory rate as a proxy measure in identifying children with non‐severe pneumonia to whom antibiotic is recommended by the IMCI guideline.

### Other common childhood illness

The widespread use of malaria RDTs is generating large numbers of children who might have previously been treated for malaria and are now being considered for an alternative diagnosis, the commonest of which is non‐severe pneumonia 12. Several studies have now documented that since the introduction of malaria RDTs, the proportion of patients treated with antibiotics has increased 14. Evidence suggests that bacteraemia is uncommon in children with non‐severe illness, as the prevalence of a positive aerobic blood culture is approximately 1% 12, 31.

### Study limitations

We did not have enough staff to assess interobserver agreement on measurements of RR, but a previous publication from the same site suggested that two independent observers only reached moderate agreement.

Secondly, temperature was only measured at the clinic, as repeated measures might have disturbed the children further at a time when they were being encouraged to settle down. However, knowledge of temperature would have provided greater insight into the results given that fever is an important factor when considering high respiratory rate.

## Conclusion

While pneumonia remains the commonest single cause of death in young children, its definition and identification of its causes continue to present a challenge to health services. In such a scenario, relatively sensitive criteria for the classification of pneumonia are important to direct management but may result in low specificity and thus misclassification. Our data show that respiratory rate tends to settle down in a calm environment and if the IMCI criteria are applied to the initial respiratory rate from the busy clinic, it may result in misclassification. Thus, to avoid unnecessary costs and implications related to antibiotic prescriptions, it is best that children be assessed in a calm environment. In addition, more studies on the use of respiratory rate as a proxy for non‐severe pneumonia diagnosis are needed and particularly, different tools that health workers can use to assess pneumonia in children are mandatory because respiratory rate is a difficult sign to measure.
